# Polymer toxicity in neurodegeneration FENIB

**DOI:** 10.18632/oncotarget.17772

**Published:** 2017-05-10

**Authors:** Noemi A. Guadagno, Elena Miranda

**Affiliations:** Department of Biology and Biotechnologies ‘Charles Darwin’, Sapienza University of Rome, Rome, Italy

**Keywords:** serpins, protein polymerisation, oxidative stress, dementia, endoplasmic reticulum

Many neurodegenerative conditions, including Alzheimer's, Parkinson's and Huntington's diseases, the prion encephalopathies and amyotrophic lateral sclerosis, are now recognised as protein conformational diseases, an ample group of pathologies characterised by the transition of wild type or mutated proteins to aggregation-prone conformations. This leads to their intracellular and/or extracellular accumulation, with adverse effects that depend on the type of protein and the site of deposition. The dementia FENIB (familial encephalopathy with neuroserpin inclusion bodies) is a rare and fatal neurodegenerative disease characterised by the presence of neuronal inclusion bodies made up of mutant neuroserpin polymers [1]. FENIB is one of the serpinopathies, a subtype of protein conformational disease with unique characteristics: mutant serpins reach a near-folded conformation and undergo ordered polymerisation within a precise location, the endoplasmic reticulum (ER) [2]. Serpin polymers do not elicit the canonical response to accumulation of misfolded proteins within the ER, the unfolded protein response (UPR), but instead activate NFkB (nuclear factor kappa-light-chain-enhancer of activated B cells) in the cytosol, probably through a leakage of calcium from the ER, a signalling pathway that has been called ‘ordered protein response’ [3]. In the dementia FENIB, increasing tendency to polymer formation by diverse mutant variants correlates with increasing severity of the associated neurodegeneration [4], and with increasing polymer formation and lower secretion in cell culture models of disease [5]. *In vivo*, polymer deposition leads to a decrease in locomotor performance in flies that overexpress mutant neuroserpin [5] and to a neurodegenerative phenotype in transgenic mice [6]. Despite the different cell and animal models developed so far, the exact mechanism of cell toxicity elicited by neuroserpin polymers remains unexplained. We have recently found that one component of this toxicity is the induction of oxidative stress. In [7], we have developed a disease model system consisting in mouse neural progenitor cells from foetal cortex differentiated *in vitro* to a neuronal phenotype, which showed correct handling and secretion of wild type neuroserpin and intracellular accumulation of G392E neuroserpin polymers in the ER, as seen in FENIB patients. In this cell model system, an RNA sequencing comparison of G392E neuroserpin and control cells has revealed the upregulation of several anti-oxidant genes in the former cells, which suggests the activation of an adaptive response to cope with the presence of neuroserpin polymers within the ER. Although in basal conditions these cells seemed as healthy as wild type neuroserpin or GFP expressing cells, G392E neuroserpin cells were more susceptible to apoptotic cell death when challenged with inhibitors of two major anti-oxidant defences: glutathione and catalase. Interestingly, there were no indications of increased oxidative stress in cells overexpressing a truncated version of neuroserpin (delta neuroserpin), which is a bona fide misfolding protein able to activate the UPR [3]. These findings provide the first evidence for the involvement of oxidative stress in the dementia FENIB, in line with other types of neurodegeneration. They also provide a basis for the observation of higher amounts of inclusion bodies in aggressive forms of FENIB, in which neurological symptoms appear earlier in life: young neurons, which cope better with oxidative stress, are less sensitive to polymer accumulation and thus a higher burden of polymers is reached before developing the disease, while lower amounts of inclusion bodies are enough to cause neuronal death and start neurodegeneration in older brains with slower polymer-forming mutant variants of neuroserpin. The exact nature of the oxidative insult caused by the accumulation of neuroserpin polymers within the ER needs further investigation, which can also contribute to a better knowledge on the role of this organelle in neurological disorders involving alterations of ER homeostasis, as seen in Alzheimer's disease, multiple sclerosis and prion diseases among others [8].

## References

[1] Davis RL (1999). Nature.

[2] Roussel BD (2011). The FEBS Journal.

[3] Davies MJ (2009). Journal of Biological Chemistry.

[4] Davis RL (2002). The Lancet.

[5] Miranda E (2008). Human Molecular Genetics.

[6] Galliciotti G (2007). Neurobiology.

[7] Guadagno NA (2017). Neurobiology of Disease.

[8] Roussel BD (2013). Lancet Neurology.

